# Supplementary material for: The cost and health-related quality of life of stroke management and care of acutely hospitalized cases in Mozambique
Source: PLoS One. 2025 Jul 24;20(7):e0328823. doi: 10.1371/journal.pone.0328823 (PMC12289051; doi:10.1371/journal.pone.0328823)
Supplement: Attachment — Submitted filename: Responses to Reviewers.docx [file pone.0328823.s002.docx]

Dear Editor

Thank you for giving us the opportunity to submit our revised manuscript titled: **The cost and quality of life of stroke management and care of acutely hospitalized cases in Mozambique** to **PlosOne.** We appreciate the time and effort that you and the reviewers have dedicated to providing your valuable feedback on my manuscript. We are grateful to the reviewers for their insightful comments on our paper. We have been able to incorporate changes to reflect the suggestions provided by the reviewers. We have highlighted the changes within the manuscript.

Here is a point-by-point response to the reviewers’ comments and concerns.

**Comments from Reviewer 1**

1) **With a sample size of 50 patients from a single urban hospital, the study's findings might have limited generalizability to the wider Mozambican population or other low-income countries. The manuscript does not sufficiently address this limitation or discuss how the findings might or might not reflect the situation in different settings within Mozambique or similar countries.**

**Response:** Thanks a lot for this comment, there is very few studies in sub-Saharan Africa assessing the cost of stroke management and/or the recovery trajectory of health-related quality of life after a stroke event, However, we have added more information and references in the discussion regarding how the findings reflect the findings in similar countries.

2) **The manuscript does not sufficiently detail the selection process for the study's participants or how potential biases were mitigated. For a study of this nature, these aspects are key to validate the findings.**

**Response:** We have included more details regarding to the selection process and the bias mitigation process: “Eligible participants were identified through daily hospital registries to avoid selection bias”. Please see lines 93-102.

3) **A comparison with findings from other regions, especially those with similar socioeconomic settings, would have provided a richer understanding of the study's implications.**

**Response:** Thanks a lot for this comment, we have now included more information and references in the discussion regarding how the findings reflect the findings in similar countries. (comment similar with the first comment). Please see line 264-279.

**Comments from Reviewer 2**

**Abstract**
**1) Results. What do the authors mean by the “informal sector”? This has not been presented anywhere else in the abstract**.

**Response:** Thanks for this comment we have included more information regarding to informal sector and we have reworded it as informal employment to make clearer throughout the manuscript. “Loss of productivity due to stroke which varied by employment status (informal, formal, pensioner and unemployed)”. Please see line 40. Formal and informal employments have also been defined on lines 107-111.

**2) Results. It would be good to report the EQ-5D results in the abstract rather than just a narrative summary.**

**Response:** We have added this information in the abstract: “The mean EQ-5D index and VAS scores of stroke patients were 0.514 (SD, 0.298), and 49.39 (SD, 20.95), respectively. Anxiety/depression 82.5% and Pain/discomfort 72.5% were the most frequently reported issues.”  See line 54.

**3) Results. Possible typo? The IQR of $13,558 cannot be 4,3 to 7,42.**

**Response:** Thanks for raising this point, it was typo and we have corrected it accordingly on line 49

**4) Conclusion. The authors should rewrite this section. What do they mean by the cost implications … is particularly high? What is the government/health care sector to do? In addition, strokes might occur at younger ages in Mozambique than say in Western Europe, but their life expectancy is also generally lower**.

**Response**: We have rewritten the conclusions in the abstract as recommended. The revision now reads: “The economic cost of stroke management in low-income sub-Saharan African countries such as Mozambique is substantially high, with considerable out-of-pocket spending, poor survival rate and a compromised health-related quality-of-life. Health system reforms designed to mitigate the individual to societal burden imposed by stroke are required.” Please see line 58

**Methods**
**1) Line 109. Details have to be provided of what the human capital approach entails.** **Response:** We provided more details regarding the human capital approach “that is, the value of lost productive time due to acute illness and short-and-long-term disabilities” Please see line 122.

***Reference:*** J. Pike and S. D. Grosse, “Friction Cost Estimates of Productivity Costs in Cost-of-Illness Studies in Comparison with Human Capital Estimates: A Review,” *Applied Health Economics and Health Policy*, vol. 16, no. 6. Springer International Publishing, pp. 765–778, Dec. 01, 2018. doi: 10.1007/s40258-018-0416-4. Please sea line 113.

**2) Line 120. The authors collected income data from patients (line 99). Why was this information not used rather than the minimum wage?**

**Response:** Thanks a lot for this recommendation, we agree, and have used the income data from the patients instead of minimum wage to compute lost productivity. Please see line 143
**3) Line 132. Why was the 3L version used over the 5L?**

**Response:** Thanks for this question. The EQ-5D-3L was selected instead of the EQ-5D-5L due to unavailability of the EQ-5D-5L in Portuguese version at the time designed the study. Please see line 159

**4) Line 132. Which valuation set, and from which country, was used to value responses to the EQ-5D?**

Response: Thanks a lot, we specified the valuation set we used: “There is no value of set for EQ-5D for both 5L and 3L for Mozambique, because of that we used the coefficients from published 3L version TTO (time trade-off) valuation study from Zimbabwe.” Please see line 160

***Reference:*** Jelsma J, Hansen K, De Weerdt W, De Cock P, Kind P. How do Zimbabweans value health states? Popul Health Metr. 2003 Dec 16;1(1):11. doi: 10.1186/1478-7954-1-11. PMID: 14678566; PMCID: PMC317383.

**5) Line 139. Why were not means presented too? Means might be useful to policy makers to establish overall costs of a disease (i.e. mean cost of stroke X number of strokes). Median costs do not allow for these calculations.**

**Response**: Thanks for this comment, we agree, and have included means on the analysis and results. Please see line 167

**6) Line 144. Why were summary measures of EQ-5D not presented? Related to this, how did the authors come up with the EQ-5D groupings?**

**Responses**: We have included summary measures of EQ-5D as recommended. Please see line 166 and Table 3.

**Results**
**1) Line 161. Why were the other 30 patients not recruited? Were not incident strokes, refused, etc…?**

**Response:** We have now clarified this in the results section. Specifically, have added on line 170 that “In total 80 stroke patients were admitted during the study period, of those, 50 were recruited (28 women, 56%) with diagnoses of first in life stroke incident, other 30 participants didn’t meet the inclusion criteria’s (20 recurrent stroke, 4 were transferred and 6 died before the informed consent).” See Line 194

**2) Line 163. I did see no mention of formal/informal employment in the methods. What do they mean by informal employment?**

**Response:** We have now included the definition of formal/informal employment in the methods section as “informal employment’ refers to employment, which is not regulated, or which does not enjoy core social protections, formal employment is regulated with social protections” on line 115

**3) Line 165. Details of the minimum wage should be given in the methods. What does the minimum wage use represent: per day, per week, per month?**

**Response**: The minimum wage represents the one-month salary we included more details in the manuscript. Please see line 199

**4) Table 1. Some elements of the table are not aligned (i.e. gender).**

**Response:** Table revised, and elements aligned. Please Table 1.

**5) Table 1, line 162. Why does the table report mean age whilst the text reports median age?**

**Response:** We have standardized age using mean age in both table and text. Please see line 197

**6) Table 1. Some abbreviations in the table need spelling out (e.g. HTN for hypertension).**

**Response:** Abbreviated word has been spelt out as recommended in the table. Please see Table 1

**7) Line 174. The authors mention that 5 patients had minor limitation, whereas the table only reports no, left, or right limitation.**

**Responses:** Thank you for these comments, we have clarified this in the text. See Line 209

**8) Paragraph starting line 185. 4 costs are presented, one is the total, and two others representing transport and medication. What did the other cost relate to?**

**Response:** We have included information regarding to the other cost on the paragraph “The total cost for the patient was $US61.55 (2,461.80MZN) corresponding to: medication $US17.85 (1,089.00MZN), transport to and from hospital $US14.13(862.05MZN) and other costs $US8.37 (510.75.MZN).” please see line 220.

**9) Paragraph starting line 189. A) Were these total costs or costs per patient?**

**Response**: This is costs per patient and have been clarified on line 229.

**B) Either costs are reported in US $ first or in MZN.**

**Response:** Thanks for rising this point, all costs have been reported in US$ with MZN reported in brackets. See line 225

**C) What were indirect outpatient care costs?**

**Response:** We have included more information related to outpatient costs: “The total Indirect outpatient care costs were $US1,520.00 (91,200.00 MZN), with $US249.18(15,199.98MZN) per patient for formal employment and $US1,229.17 (73,750.00MZN) with $US43,36 (2,644.96MZN) per patient for informal employment”. Please see line 232

**10) Table 2. Much of the details in Table 2 should be reported in the methodology.**

**Response:** Thanks for this comment, we have revised the method to include details of Table as recommended. Please see line 112

**11) Figure 2. I’m not sure how results of the EQ-5D are reported in Table 2. Are these the people who said I have some or extreme problems in each of the 5 domains?**

**Response:** Thank you for this comment, we have improved the graphic to include all 3 levels of the 5 dimensions. Please see figure 328

**12) In the methods the authors reported that EQ-5D results were presented in 4 groups. No such data are presented in the results.**

**Response:** We have now reported the EQ-5D results in the table 3

**Discussion**
**1) Line 229. The authors appear to imply that having a stroke young, as is the case in Mozambique with average age at first stroke being 60 years, will result in an enormous burden for the years to come. However, I note from the World Bank that the average life expectancy in Mozambique is 61.17 years, the age at which patients are having their strokes. Therefore, I do not think this line of argument hold. By contrary, Italy where the authors quote estimates of 74.9 years as the age of first stroke, life expectancy is 82.34 years.**

**Response:** Thanks for this comment, we have revised our arguments regarding to age of stroke. Please se line 286

**2) Limitations. I found this section very short. Chief amongst the limitations of this study is the small patient sample size. Also, how generalizable are their results to the rest of the country? For example, I note that the study was based on a tertiary hospital in the capital.**

**Response:** We have included more information in this section: “Despite the strength of our study, there are some limitations that need to be acknowledged. Firstly, unlike many advanced countries with structured administrative hospital and cost data, no detailed cost structure is attributed to care provided in Mozambique health facilities for the financial management of medical and paramedical activities. Also, the unit costs charged by the national health service are highly subsidized. Hence, the estimates in this study are patient-reported and could be subject to recall bias. Secondly, this study was implemented in only one tertiary health facility with limited number of patients being admitted during the study period, hence, our findings should be interpreted with caution.” Please see line 316.
